# Exploring GNRA tetraloop-like motifs in nucleic acid 3D structures

**DOI:** 10.1038/s41598-025-21072-9

**Published:** 2025-10-23

**Authors:** Janusz M. Bujnicki, Eugene F. Baulin

**Affiliations:** 1 International Institute of Molecular and Cell Biology in Warsaw, Warsaw, Poland; 2 IMol Polish Academy of Sciences, Warsaw, Poland

**Keywords:** GNRA tetraloop, RNA structure, 3D conformation, Motif search, Backbone topology, RNA–protein interactions, Biochemistry, Computational biology and bioinformatics, Structural biology

## Abstract

Structured nucleic acids play key roles in a variety of molecular mechanisms and have numerous applications in medicine and biotechnology. Stem-loops with specific tetraloop sequences, such as UNCG and GNRA, are among the most well-studied recurrent building blocks of structured RNAs, known to adopt very stable structures. The GNRA tetraloop motif frequently engages in long-range interactions with specific RNA receptors and can also be recognized by proteins. While several examples of GNRA-like conformations have been reported in non-tetraloop strands, no systematic survey has been conducted to explore the landscape of these variants. In this work, we report a comprehensive survey of GNRA tetraloop-like motifs within known 3D structures of nucleic acids, based solely on isostericity and without imposing restraints on sequence, loop type, backbone topology, or interactions involved. We identified twelve recurrent backbone topology variants of the motif, including all five possible two-strand variants and six three-strand variants. Among the pentaloop variants, we observed four different locations of the looped-out residues, with three of them directly interacting with proteins. These GNRA-like motifs highlight the versatility of favorable RNA 3D conformations to fit in diverse backbone contexts and modulate intermolecular interactions, which can be leveraged in RNA motif design.

**Supplementary Information:**

The online version contains supplementary material available at 10.1038/s41598-025-21072-9.

## Introduction

Structured nucleic acids play key roles in a variety of molecular mechanisms^1–3^ and have numerous applications in medicine and biotechnology^4,5^. The 3D structure of nucleic acids is organized hierarchically, comprising recurrent building blocks such as base pairs, helical segments, and tertiary motifs, with tetraloops of specific sequence patterns among the most prevalent^6–8^. GNRA tetraloops (N stands for aNy nucleotide, R stands for puRine) are overrepresented in non-coding RNAs, including ribosomal RNAs, ribozymes, and riboswitches^8,9^. They adopt very stable structures in both RNA and DNA variants^10^.

**Fig. 1:**
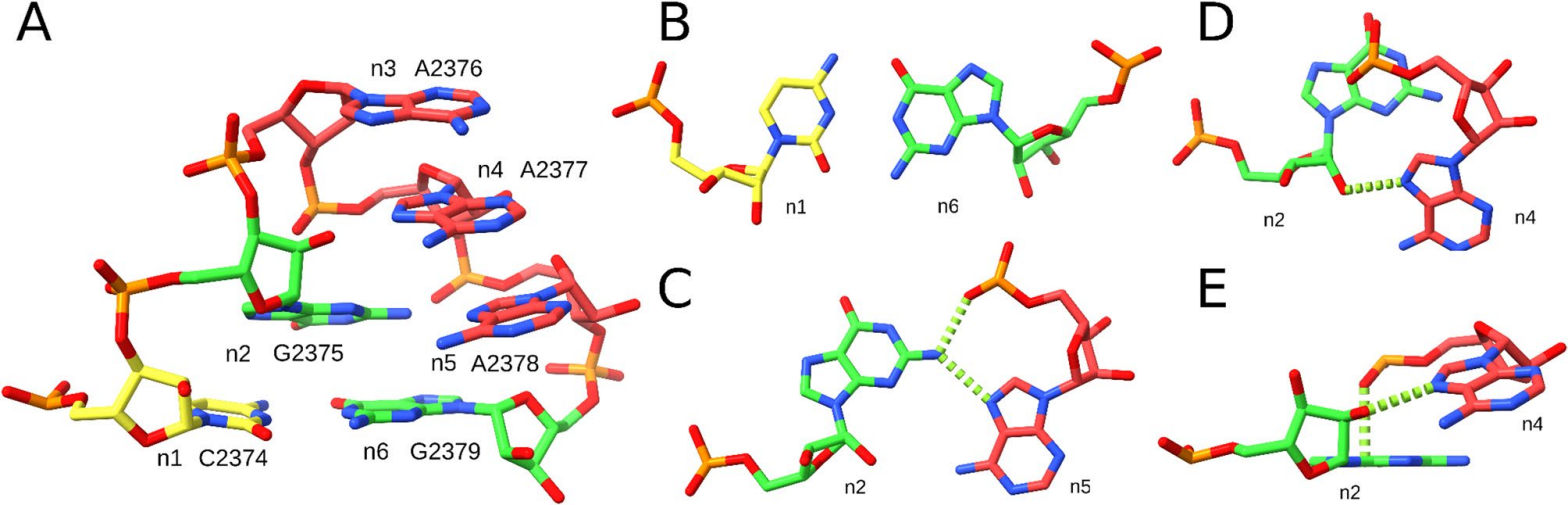
Reference instance of the GNRA tetraloop motif. (**A**) Residues n1-n6 (PDB entry 8VTW, 23S rRNA, chain 1A, residues 2374–2379), (**B**) Flanking canonical C-G base pair between residues n1 and n6. (**C**) tSH G-A base pair between residues n2 and n5, and the G-RpA base-phosphate interaction. (**D**) Top and (**E**) side views of the R-rG base-ribose hydrogen bond and the NpR-G oxygen-base stacking interaction between residues n2 and n4. The color scheme shows guanosines in green, adenosines in red, cytidines in yellow, and uridines in blue (not present in this figure). Dashed green lines indicate key interactions. The figure was prepared using UCSF ChimeraX (https://www.cgl.ucsf.edu/chimerax/).

The characteristic structural features of the GNRA tetraloop motif include the trans-Sugar-Hoogsteen (tSH) G-A base pair, G-RpA base-phosphate hydrogen bond, R-rG base-ribose hydrogen bond, and NpR-G oxygen-base stacking^11^ (Fig. 1). The U-turn motif (GNR) of the GNRA tetraloop arranges the stacked loop bases such that their Watson–Crick (WC) edges remain available for interactions^12,13^. Consequently, GNRA tetraloops often form long-range tertiary interactions, primarily through A-minor motifs^14–17^, for example, interactions between the L9 loop and the P5 helix of a group I intron required for its proper folding and self-splicing activity^15^. The highly specific GAAA tetraloop/11nt receptor interaction is among the most stable long-range tertiary motifs formed with a GNRA loop^17^ and has been widely used as a building block in RNA nanostructures and scaffolding^16,18^. Conserved GNRA tetraloops within viral internal ribosome entry site (IRES) elements are essential for their role in translation^19–21^. The universally conserved sarcin-ricin loop (SRL) of the ribosome contains a GAGA tetraloop that interacts with the elongation factors EF-Tu and EF-G during translation and is specifically recognized by the ricin toxin^22,23^.

GNRA-like motifs can also be formed by non-tetraloop strands, such as pentaloops^24–26^ and hexaloops^27^. These non-tetraloop variants can also be specifically recognized by proteins, such as in the N peptide-BoxB RNA complexes of bacteriophages^24–29^. It has been shown that the GNRA sequence can be split and interrupted by other nucleotides (GN(n)/RA) and still adopt the characteristic GNRA structure capable of forming long-range interactions^30,31^. The UAA/GAN internal loop, found in 23S rRNA, RNase P, and group I and II introns^32^, falls within this extended consensus (GAN/UAA in reverse order of strands) and is also considered a GNRA tetraloop-like motif^33,34^. Similar to GNRA tetraloop/receptor motifs, UAA/GAN internal loops form cross-strand AAA stacks involved in long-range A-minor interactions^32^. GNRA-like motifs can even mediate RNA–ligand interactions, for example, in the complex of an in vitro selected ATP-binding aptamer with AMP, where the ligand intercalates beneath the R residue of the GNR strand^35^. Moreover, GNRA-like motifs can be formed by non-GNRA sequences^17,36,37^, such as UMAC loops (where M is A or C), found in ribosomes and BoxB RNAs of bacteriophages^27,36,38^. While several previous studies have surveyed the structural landscape of tetraloops and larger hairpins^36,39^, the GNRA tetraloop motif has not been explored in a backbone topology-independent manner. As a result, the diversity of known GNRA-like motif topologies remains limited to a few empirical observations^35,40–43^.

In this work, we conducted an exhaustive analysis of the GNRA tetraloop motif instances across known 3D structures of nucleic acids, without imposing any restraints on sequence, loop type, backbone topology, or involved interactions. We observed four distinct insertion sites in the pentaloop variants of the motif, with three of them forming interactions with proteins. We identified twelve recurrent backbone topology variants of the GNRA structure, including all five possible two-strand variants and six out of ten theoretically possible three-strand variants. These GNRA-like motifs illustrate the remarkable capacity of favorable RNA 3D motifs to fit in diverse backbone contexts, adding another dimension to the complexity of RNA folding.

## Results

We defined the GNRA tetraloop motif as consisting of six residues (n1 to n6), which include the GNRA tetraloop (n2 to n5) and one flanking canonical base pair n1-n6 (Fig. 1). To explore the landscape of GNRA-like motif variants, we selected the reference instance (Fig. 1A) and searched for its matches in nucleic acid-containing PDB entries^44^ using the ARTEM tool^37^, see the Methods section for details.

### Dataset overview

We identified 23,283 non-redundant GNRA tetraloop matches comprising three to six residues across 3122 PDB entries (Supplementary Table S1), representing 13,729 structurally unique motifs (Supplementary Table S2). These matches were found in 86 non-coding RNA families from Rfam^45^ (53% of all families with 3D structures available in the PDB) and 1063 classes from the BGSU representative set of RNA structures^46^ (22.6%), with only 22 matches involving DNA residues. The list of Rfam families included 21 riboswitch aptamers, 19 ribozymes, 19 viral RNAs, including 4 IRES elements, 9 spliceosomal RNAs, and 8 ribosomal RNAs. Among the most frequently observed modifications were PSU (199 matches), A2M (162), MA6 (133), OMG (104), and OMC (97). 10,894 hits matched all six residues of the reference. The n1 and n6 positions were identified as the least constrained, with 4068 and 6061 unmatched cases, respectively, followed by n5 (3038) and n3 (2879). Notably, among the n2 and n4 residues, both critical for the GNRA tetraloop motif and the U-turn motif, the n2 was found unmatched nearly five times more often than n4 (2393 vs. 521 cases, respectively).

**Fig. 2:**
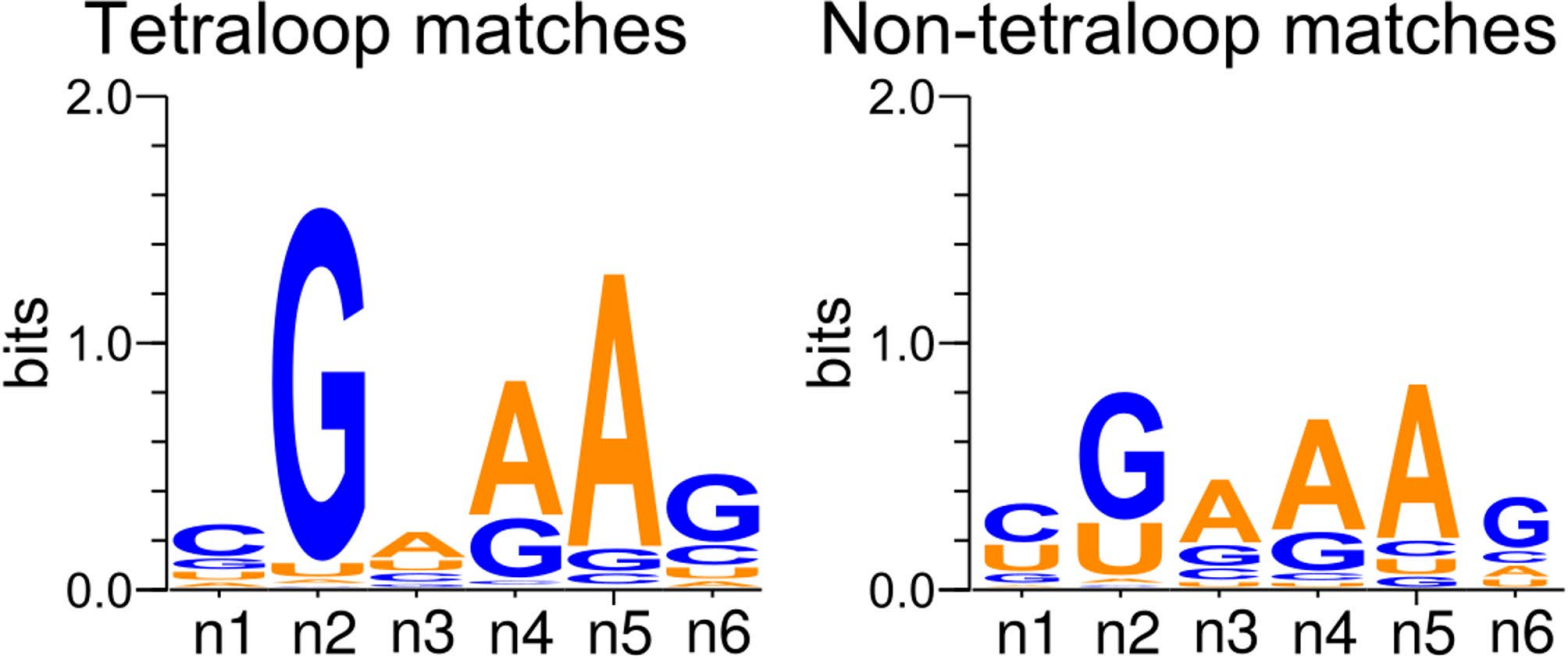
Motif logo of tetraloop and non-tetraloop matches of the GNRA tetraloop motif. The left logo shows the 4,881 tetraloop matches with a single continuous n1-n6 strand. The right logo shows the remaining 18,402 matches. The figure was prepared using WebLogo 3 (https://weblogo.threeplusone.com/create.cgi).

The n2 and n5 residues were identified as the most conserved positions, with 72% and 78% of the matched bases being G and A, respectively (Fig. 2). The n4 position matched a purine in 80% of cases, and, notably, adenosines accounted for 57% of the matched n3 residues. 4881 hits with matched n2-n5 residues were formed by tetraloops, 1409 by pentaloops, 809 by hexaloops, and 2303 by longer hairpin loops. The remaining instances included 5050 matches with non-hairpin strands and 8831 incomplete matches with at least one unmatched residue from n2 to n5.

Of the 23,283 matches, 768 (3.3%) were involved in forming crystal contacts, with n3 (610 instances), n4 (416 instances), and n5 (341 instances) being the most frequently interacting positions. This aligns with the known propensity of the GNRA tetraloop motif to form long-range interactions, as in the GAAA/11nt tetraloop-receptor motif^16^. However, ARTEM did not identify any matches spanning multiple asymmetric units, i.e., including crystal contacts within the motif residues. Next, we analyzed instances spanning multiple RNA chains within a single unit. We observed only 170 (0.7%) multi-chain variants (166 two-chain and 4 three-chain matches), 27 of which were complete six-residue matches. These included two three-chain variants from a synthetic DNA nanostructure (Supplementary Fig. S1A), 18 RNA variants with n1 and n2 residues from one chain and residues n3 to n6 from another chain (Supplementary Fig. S1B), five two-chain variants of two types from mitochondrial rRNAs (Supplementary Fig. S1C, D), and two instances from FMN riboswitches (Supplementary Fig. S1E). In the latter case, the FMN riboswitch is represented by two fragments, chains X and Y, with the n5 adenosine of chain X intercalating between the n4 and n6 residues of a triloop from chain Y. Finally, we surveyed instances that span distant stems and loops within a single RNA chain. A total of 219 (0.9%) single-chain matches featured at least one long-range interaction, defined as a pair of residues from distant RNA secondary structure elements. These included 151 five-residue instances with an unmatched n6 residue and a long-range n4-n5 pair, and 24 six-residue cases resembling the multi-chain FMN riboswitch match. Thus, the vast majority of GNRA-like matches involved locally arranged residues, both in terms of RNA sequence and RNA secondary structure.

**Fig. 3:**
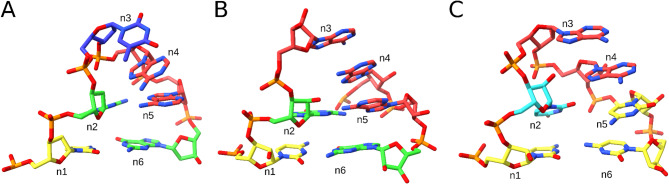
Known GNRA-like motif variants captured by ARTEM. (**A**) DNA form, GTAA tetraloop, 1.04 Å RMSD, PDB entry 2N8A, chain B, residues 9–14. (**B**) UAA/GAN internal loop, 1.25 Å RMSD, PDB entry 4V72, 23S rRNA, chain BA, residues 1376–1378 and 1353–1355. (**C**) CUAACC hexaloop, 1.47 Å RMSD, PDB entry 1NYB, phage φ21 boxB RNA, chain B, residues 10–15. The color scheme shows guanosines in green, adenosines in red, cytidines in yellow, and thymidines and uridines in blue. The figure was prepared using UCSF ChimeraX (https://www.cgl.ucsf.edu/chimerax/).

ARTEM successfully captured known variants of the GNRA tetraloop motif. Among others, the dataset included the DNA form of the motif from a gapped dumbbell DNA^47^, which retained all characteristic interactions except the n2-n4 R-rG base-ribose hydrogen bond (Fig. 3A); the UAA/GAN internal loop, which lacked the n2-n5 G-RpA base-phosphate interaction and the n2-n4 NpR-G oxygen-base stacking, instead forming an n2-n4 G-NpR base-phosphate hydrogen bond (Fig. 3B); and the UMAC structure formed by the CUAACC hexaloop of the bacteriophage φ21 boxB RNA in complex with the N peptide^27^, which lacked the n2-n5 tSH base pair and featured a non-canonical cWW C-C base pair as the flanking n1-n6 interaction (Fig. 3C).

Among the 13,729 unique motifs, 236 were identified in at least three different Rfam families and three different BGSU representative classes and can therefore be considered recurrent. The GAAA tetraloop, which did not form long-range base pairs, was found in eight Rfam families with a G-C flanking n1-n6 base pair, including group II introns, SAM and Glutamine riboswitches, and bacterial RNase P, and in seven families with a C-G flanking base pair, including aptamers of Lysine, Guanidine-I, and AdoCbl-II riboswitches. The GUGA tetraloop, in which the n4 and n5 residues formed long-range base pairs, was identified in six Rfam families with a G-C flanking n1-n6 base pair, including the glmS ribozyme and ribosomal RNAs. All other motifs were found in at most five Rfam families, with a UAA/GAN six-residue match, identified in 21 representative classes across three families of ribosomal RNAs, being the most widespread non-hairpin motif.

### Pentaloop variants

**Fig. 4:**
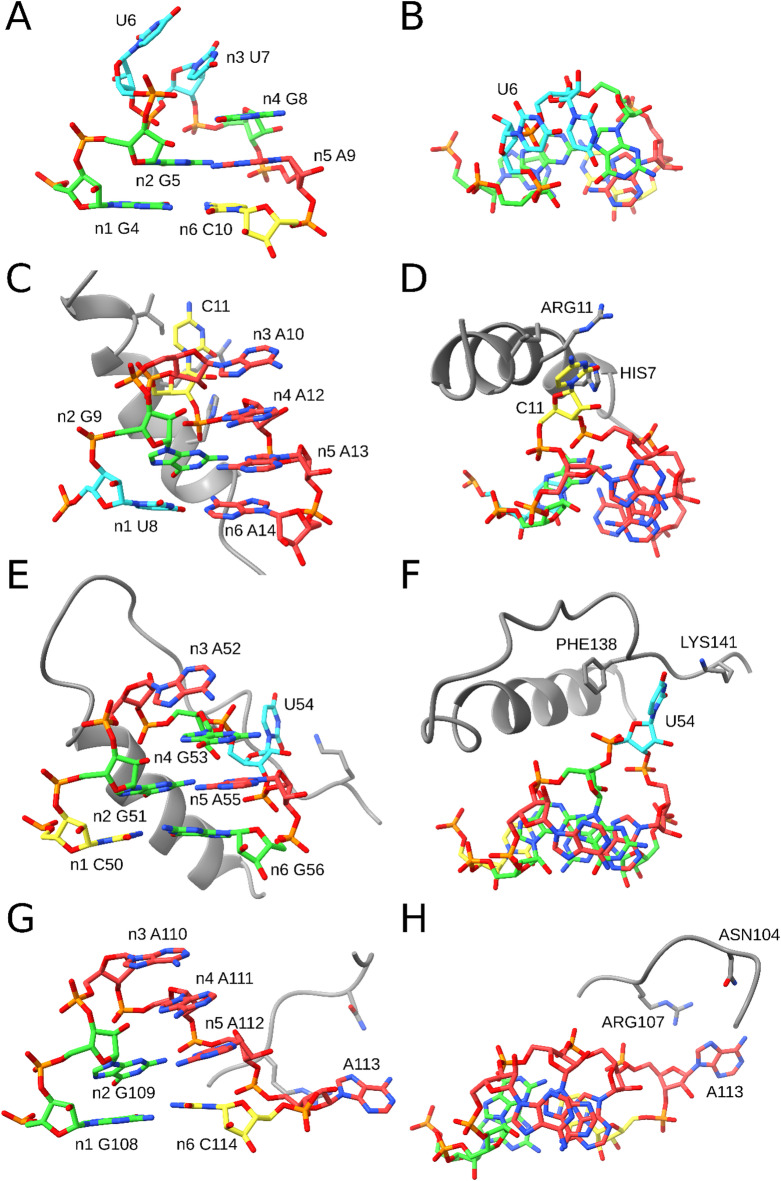
Pentaloop variants of the GNRA tetraloop motif captured by ARTEM. The left column is a side view, and the right column is a top view. (**A**, **B**) n2-n3 insertion variant from a viral tRNA-like structure, 1.28 Å RMSD, PDB entry 7SC6, chain C. (**C**, **D**) n3-n4 insertion variant from the phage P22 N peptide-BoxB RNA complex, 1.15 Å RMSD, PDB entry 1A4T, chain A. (**E**, **F**) n4-n5 insertion variant from a CRISPR RNA, 0.84 Å RMSD, PDB entry 6LNB, chain M. (**G**, **H**) n5-n6 insertion variant from an RNase MRP structure, 1.03 Å RMSD, PDB entry 6W6V, chain A. The color scheme shows guanosines in green, adenosines in red, cytidines in yellow, and thymidines and uridines in blue. The interacting proteins are shown in gray. The figure was prepared using UCSF ChimeraX (https://www.cgl.ucsf.edu/chimerax/).

We analyzed the identified pentaloop variants of the GNRA tetraloop motif. With ARTEM, we did not detect any pentaloop variants with an insertion between n1 and n2 but identified all four of the other possible insertion variants (Fig. 4). We selected non-ribosomal matches with the lowest RMSD to the reference as representative instances. The n2-n3 insertion instance (Fig. 4A, B) was found in a viral tRNA-like structure^48^, with an extra uracil stacked on top of n3, and did not form any external interactions. The three remaining representatives interacted with proteins.

The n3-n4 insertion variant was found in the N peptide-BoxB RNA complex of bacteriophage P22^24^ (Fig. 4C, D), with a cytosine looped out from the GACAA pentaloop and interacting with arginine ARG11 (O2-NE distance of 3.3 Å) and histidine HIS7 (O2-NE2 distance of 3.6 Å) of the N peptide. The n4-n5 insertion variant was found in a CRISPR RNA structure^49^ (Fig. 4E, F). The U54 residue was looped out and formed a stacking interaction with phenylalanine PHE138 (~ 5 Å distance between the ring planes) and a hydrogen bond with lysine LYS141 (O2-NZ distance of 4.2 Å) of the Cas6 protein. The n5-n6 insertion variant was observed in an RNase MRP structure^50^ (Fig. 4G, H). The looped out adenosine A113 was exposed to arginine ARG107 (N7-NH2 distance of 4.5 Å) and asparagine ASN104 (N6-NO2 distance of 5.7 Å) of the ribonucleases P/MRP protein subunit POP1.

### Backbone topology landscape of GNRA-like motif variants

**Fig. 5:**
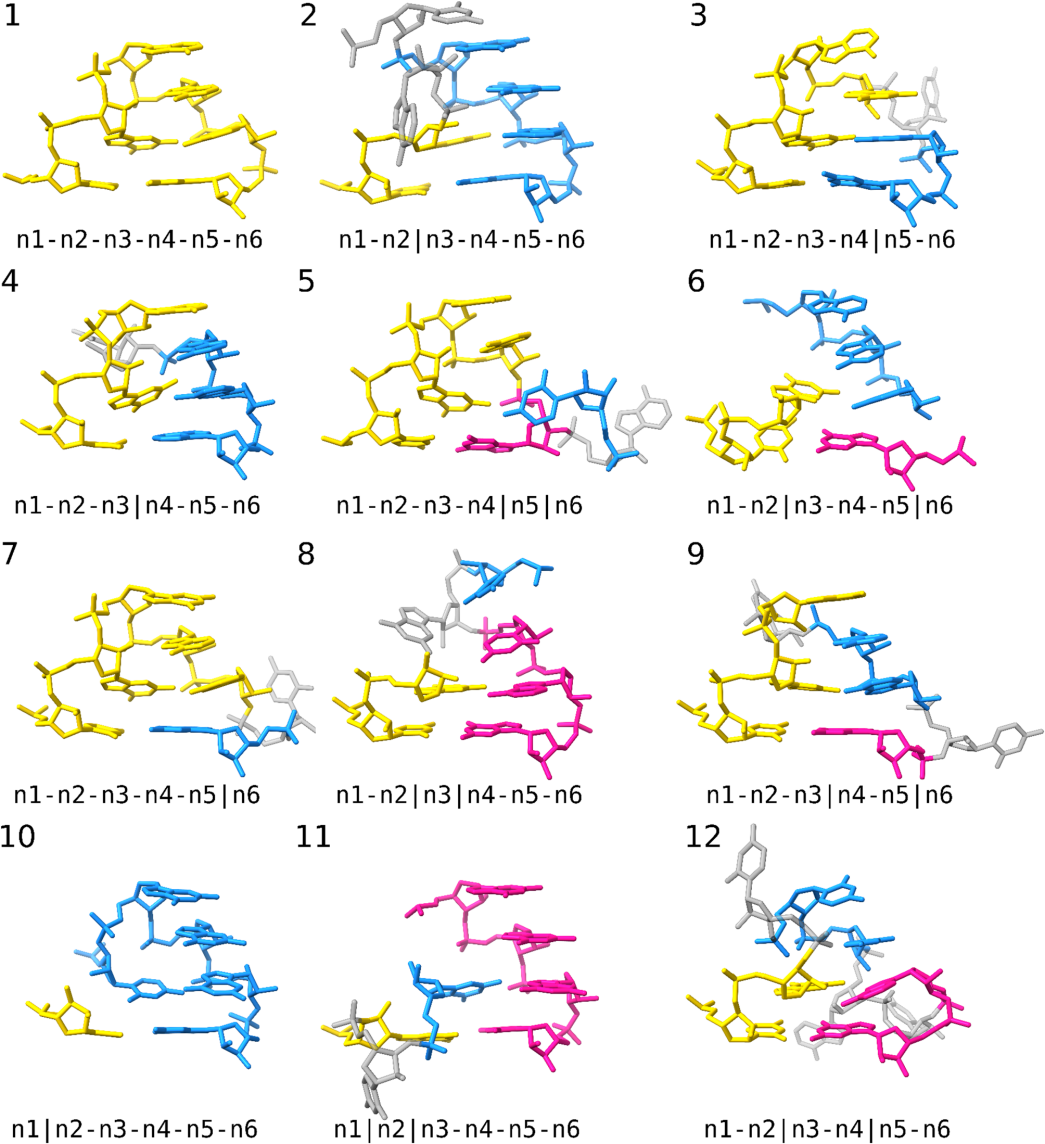
Recurrent backbone topology variants of the GNRA tetraloop motif identified by ARTEM. Continuous strands of the motif are shown in gold, blue, and pink. Looped-out residues are shown in gray. The figure was prepared using UCSF ChimeraX (https://www.cgl.ucsf.edu/chimerax/).

We classified all 13,729 motifs into 59 distinct backbone topologies (Supplementary Table S3), based on binary relationships between neighboring positions in the GNRA tetraloop motif. For example, in the canonical GNRA tetraloop, all neighboring positions are consecutive residues, yielding the backbone topology “n1-n2-n3-n4-n5-n6”. In contrast, in the UAA/GAN motif, positions n3 and n4 are not consecutive in the RNA sequence, resulting in the “n1-n2-n3|n4-n5-n6” topology. In total, we identified 17 distinct topologies among the six-residue matches, out of the 2^5^ = 32 possible variants; 12 of these were found in at least three BGSU classes and three Rfam families (Fig. 5, Table 1). We then analyzed representative motifs from each variant, selecting those with the lowest RMSD to the reference. When a representative motif contained artifacts, for example, overlapping residues in low-resolution entries, we selected the next-best suitable match.

**Table 1:** Representative motifs of the recurrent backbone topology variants of the GNRA tetraloop motif.

**#**	Topology, #strands	PDB entry, chain ID	Residues n1-n6	#Rfam, #BGSU	n4-n2 atom-base stacking	n4-n2 base-ribose	n2-n5 BPh	n2-n5 base pair	n1-n6 base pair
1	n1-n2-n3-n4-n5-n6 1	8VTW 1A	C2374, G2375, A2376, A2377, A2378, G2379	49 401	OP2-G	AN7-GO2′ 2.7 Å	GN2-OP2 2.7 Å	G-A tSH	C-G cWW
2	n1-n2|n3-n4-n5-n6 2	8BTD St	C289, G290, G275, G276, A277, G278	19 143	OP2-G	GN7-GO2′ 3.9 Å	GN2-OP2 2.9 Å	G-A tSH	C-G cWW
3	n1-n2-n3-n4|n5-n6 2	6LNB M	C50, G51, A52, G53, A55, G56	13 76	OP2-G	GN7-GO2′ 3.7 Å	–	G-A tSH	C-G cWW
4	n1-n2-n3|n4-n5-n6 2	4V5H AA	U1165, G1166, A1167, A1169, A1170, A1171	10 125	OP2-G (U1168-n2)	AN6-GO2′ 2.9 Å	GN2-OP2 3.1 Å	G-A tSH	U-A cWW
5	n1-n2-n3-n4|n5|n6 3	5WE4 A	C1752, G1753, A1754, A1755, U1758, G1756	6 63	OP2-G	AN7-GO2′ 2.8 Å	GN2-OP2 3.2 Å	–	C-G cWW
6	n1-n2|n3-n4-n5|n6 3	4V6W A5	C1355, G1356, A1151, A1152, G1153, G1331	5 32	–	AN6-GO2′ 3.6 Å	GN2-OP2 4.2 Å	G-G tSH	C-G cWW
7	n1-n2-n3-n4-n5|n6 2	1YJN 0	C1793, G1794, G1795, A1796, A1797, G1799	4 74	OP2-G	AN7-GO2′ 2.8 Å	GN2-OP2 2.8 Å	G-A tSH	C-G cWW
8	n1-n2|n3|n4-n5-n6 3	7PAU 3	C257, G258, U245, U247, A248, G249	4 27	–	UO4-GO2′ 3.2 Å	GN2-OP2 4.5 Å	G-A tSH	C-G cWW
9	n1-n2-n3|n4-n5|n6 3	7RQC 2A	C955, G956, A957, A959, A960, G962	4 26	AO2′-G (n3-n2)	AN7-GO2′ 3.1 Å	GN2-OP2 2.7 Å	G-A tSH	C-G cWW
10	n1|n2-n3-n4-n5-n6 2	8HL5 A23S	C1161, C1275, G1276, G1277, G1278, G1279	3 25	OP2-C	–	CN4-OP2 2.2 Å	–	C-G cWW
11	n1|n2|n3-n4-n5-n6 3	9E71 4	G307, G309, G293, G294, G295, C296	3 16	OP2-G	–	GN2-OP2 3.3 Å	G-G tSH	G-C cWW
12	n1-n2|n3-n4|n5-n6 3	6YLX 1	C2840, G2841, U2843, C2844, A2847, G2848	3 7	OP2-G	–	–	G-A tSH	C-G cWW

As expected, the canonical one-strand variant of the GNRA tetraloop motif was the most frequent motif, identified in 49 different Rfam families. It was followed by three two-strand variants with a single chain break: the n2|n3 break found in 19 Rfam families, the n4|n5 break in 13 families, and the n3|n4 variant in 10 families. The remaining two-strand variants were less common, with n5|n6 found in four (rRNAs and RNase MRP) and n1|n2 found in three Rfam families (rRNAs only). The two most frequent three-strand variants were “n1-n2-n3-n4|n5|n6” and “n1-n2|n3-n4-n5|n6”, identified in six (rRNAs, group II introns, and FMN riboswitch) and five Rfam families (rRNAs only), respectively. Representative motifs of all twelve topologies featured at least three of the five key interactions characteristic of the GNRA tetraloop motif (Table 1).

**Fig. 6:**
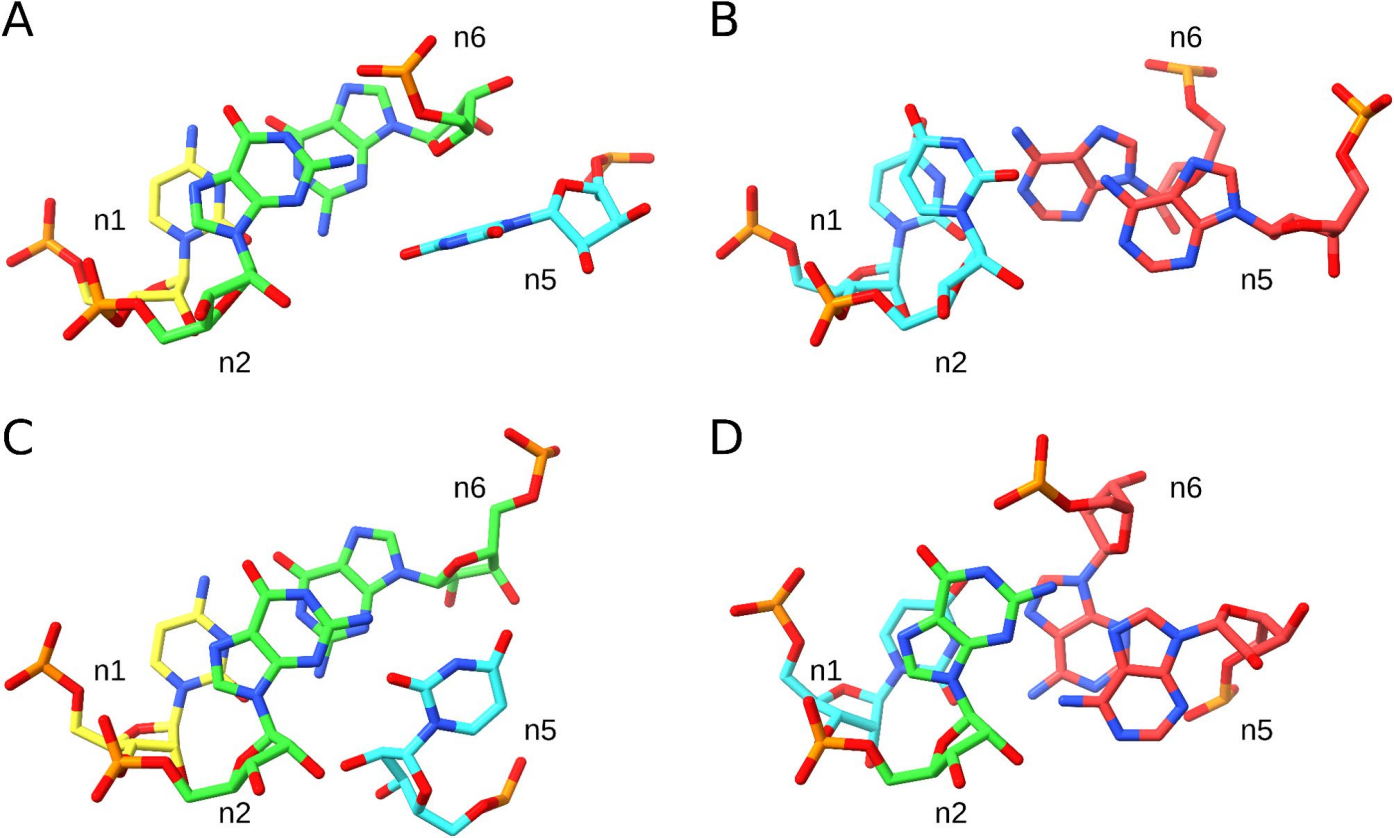
Base pair diversity of the GNRA-like topology variant n1-n2-n3-n4|n5|n6 (#5). (**A**) 23S rRNA, 0.91 Å RMSD match, PDB entry 5WE4, chain A. (**B**) Group II intron, 1.23 Å RMSD match, PDB entry 6MEC, chain A. (**C**) 16S rRNA, 1.44 Å RMSD match, PDB entry 8HL5, chain A16S. (**D**) 16S rRNA, 1.49 Å RMSD match, PDB entry 8HKZ, chain A16S. The color scheme shows guanosines in green, adenosines in red, cytidines in yellow, and uridines in blue. The figure was prepared using UCSF ChimeraX (https://www.cgl.ucsf.edu/chimerax/).

We surveyed the most populated three-strand “n1-n2-n3-n4|n5|n6” topology to assess the diversity of instances in terms of key interactions (Fig. 6, Table 2). In addition to the representative motif from 23S rRNA, which lacks an n2-n5 base pair (Fig. 6A), we identified instances featuring n2-n5 base pairs involving each of the three possible edges of the n5 residue. A match from a group II intron included U-A cSW (cis-Sugar-Watson–Crick) n2-n5 base pair and the canonical U-A n1-n6 base pair (Fig. 6B); a 16S rRNA match featured a G-U cSS n2-n5 base pair (Fig. 6C); and another 16S rRNA match displayed the classic G-A tSH n2-n5 base pair along with a non-canonical U-A tWH n1-n6 base pair (Fig. 6D). All four instances contained the other three key interactions characteristic of the GNRA tetraloop motif (Table 2).

**Table 2:** Four representative motifs of the GNRA-like topology variant n1-n2-n3-n4|n5|n6 (#5).

PDB entry, chain ID	Residues n1-n6	n4-n2 atom-base stacking	n4-n2 base-ribose	n2-n5 BPh	n2-n5 base pair	n1-n6 base pair
5WE4 A	C1752, G1753, A1754, A1755, U1758, G1756	OP2-G	AN7-GO2′ 2.8 Å	GN2-OP2 3.2 Å	-	C-G cWW
6MEC A	U766, U767, G768, A769, A248, A771	OP2-U	AN7-UO2′ 2.8 Å	UO2-OP2 3.3 Å	U-A cSW	U-A cWW
8HL5 A16S	C373, G374, A375, A376, U52, G49	OP2-G	AN6-GO2′ 2.6 Å	GN2-OP2 2.3 Å	G-U cSS	C-G cWW
8HKZ A16S	U335, G336, A337, G338, A96, A339	OP2-G	GN7-GO2′ 3.9 Å	GN1-OP2 2.4 Å	G-A tSH	U-A tWH

## Discussion

In this work, we conducted a comprehensive survey of GNRA tetraloop-like motifs without setting any restraints on sequence, interactions, or backbone topology. In addition to previously known non-canonical variants of the motif, including DNA forms, UMAC sequences, and UAA/GAN internal loops, we identified several new GNRA-like backbone topology variants. These included all possible two-strand variants, with n2|n3, n3|n4, and n4|n5 chain breaks among the most frequent, and n1|n2 and n5|n6 breaks less favored. We also identified six three-strand variants of the GNRA-like motif, including the recurrent “n1-n2-n3-n4|n5|n6” topology, which accommodates a diverse range of n2-n5 base pair geometries. Furthermore, we observed four different insertion locations in the pentaloop variants of the GNRA tetraloop motif, three of which were involved in RNA–protein interactions. Although numerous two- and three-strand variants were identified, more than 98% of the analyzed GNRA tetraloop-like matches represented local motifs in terms of RNA secondary structure, unlike, for example, A-minors, a substantial fraction of which are long-range interactions ^51,52^.

The fact that the n1|n2 chain break was identified as the least frequent two-strand variant of the GNRA tetraloop motif, and was not found among the pentaloop variants, aligns with the previously noted importance of n1-n2 connectivity in forming the motif^30^. However, the diversity of identified GNRA-like backbone topology variants suggests that the backbone acts as a contributing factor rather than a strict determinant. Overall, no definitive determinants of the GNRA structure have been identified, as it can accommodate non-GNRA sequences, one to three strands, RNA or DNA residues, and various types of core interactions. Examining the representatives of the identified GNRA-like variants (Fig. 5, Table 1), it is not obvious where to place a clear demarcation to decide what does and does not qualify as a GNRA tetraloop-like motif. In this work, the principle of the ARTEM tool, to identify matches based on motif isostericity (the 3D alignability of residues), served as the only constraint enabling such separation. In contrast, defining the GNRA tetraloop motif based on the centroid reference (Fig. 1) is straightforward. Thus, we observe that the GNRA tetraloop motif, like arbitrary RNA tertiary motifs in general, can be viewed as a clearly defined, energetically favorable gravity center surrounded by a fuzzy cloud of less favorable variants. Accordingly, for searching GNRA tetraloop-like motifs with ARTEM, we recommend using the reference instance shown in Fig. 1 along with an RMSD threshold of 1.5 Å for six-residue matches. It is important to note, however, that we do not account for the structural environment of the motif and therefore cannot distinguish between self-sufficient instances and those that depend on specific interaction partners. The ability to differentiate between these cases could, in principle, provide the desired criterion for classification.

The diversity of RNA–protein interaction modes observed among the pentaloops captured by ARTEM highlights the biological relevance of the GNRA tetraloop motif variants. In the analyzed representative pentaloop instances, the looped-out bases were directly exposed to proteins, contributing to the specificity of the intermolecular interactions. The functional significance of such structural variants of the motif was confirmed in previous studies. For example, different pentaloop and hexaloop variants of bacteriophage BoxB RNA stem-loops were shown to mediate specific binding with cognate N peptides^24,25,27^. The GAA triloop of the ATP-binding RNA aptamer was shown to facilitate RNA–ligand binding through intercalation of the ligand between the n4 and n6 motif positions^35^. Similarly, the functional relevance of different sequence variants of the GNRA tetraloop motif was demonstrated in a variety of studies, including conserved tetraloop sequences essential for viral IRES elements^19–21^, the SRL GAGA tetraloop specifically recognized by ricin toxin^22,23^, and the GAAA variant, which forms the most stable GAAA/11nt tetraloop-receptor long-range RNA–RNA interactions^16^. Thus, systematic analysis of RNA tertiary motif variability, such as the investigation of GNRA tetraloop motifs presented in this work, serves as a step toward decoding the RNA–RNA, RNA–protein, and RNA–ligand recognition modes and holds promise for modulating binding affinities through RNA motif design.

While ARTEM proves to be a powerful tool for the analysis of RNA 3D motifs, it has two notable limitations. First, it requires the user to define the RNA motif of interest. Second, its results are biased toward the selected reference motif instances. In future work, we plan to equip ARTEM with a built-in library of recurrent RNA 3D motifs, which will significantly expand the tool’s applicability. Ultimately, providing the research community with a comprehensive resource will make nucleic acid 3D structural data more interpretable and actionable for non-structural biologists.

## Methods

To survey GNRA-like motifs, we retrieved all PDB entries containing RNA, DNA, or hybrid nucleic acid molecules (18,741 entries as of January 13th, 2025, mmCIF format^44^). We retained only the first model in each entry and added interacting symmetry mates using ChimeraX^53^ (“*crystalcontacts #1 distance 6*”) to account for crystal contacts (only for entries under 10 MB with the experimental method “X-RAY DIFFRACTION”). To select the reference GNRA tetraloop instance (23S rRNA, entry 8VTW, chain 1A, residues 2374–2379)^54^, we identified the hairpin loop motif annotated as “GNRA” in the RNA 3D Motif Atlas that contained the highest number of instances^55^ (motif HL_85603.2, Hairpin Loop Motif Atlas Release 3.88). This motif comprised 284 hairpins, including 271 tetraloops, 10 pentaloops, and 3 hexaloops. We then searched for the centroid instance, defined as the one with the lowest median RMSD to other instances of the motif, using ARTEM^37^ for pairwise comparisons. The ARTEM tool superimposes two input nucleic acid structures of N and M residues using single residue-residue matches as seeds. For each of the N × M possible superpositions, ARTEM identifies mutually closest residues within a specified distance and reports the match size and RMSD if the criteria are satisfied. For seed superpositions, ARTEM uses five-atom residue representations (three base atoms, one ribose atom, and one phosphate atom), while for superimposing matches and calculating RMSD, it employs 3-atom base representations.

We then searched for matches of the GNRA reference in the PDB entries using ARTEM in two modes: (i) using the six-residue reference (n1 to n6, GNRA plus the flanking base pair), counting matches of five residues with an RMSD under 1.25 Å and six residues with an RMSD under 1.5 Å; (ii) using the four-residue reference (n2 to n5, GNRA only), counting matches of three residues with an RMSD under 0.75 Å that include the n2 residue and matches of four residues with an RMSD under 1.0 Å. The RMSD thresholds were defined based on pairwise RMSD distributions between instances of the HL_85603.2 motif (Supplementary Fig. S2). The two-mode approach was designed to avoid numerous trivial two-base-pair hits (the G-A base pair plus the flanking base pair) that would be identified by ARTEM if searched with the six-residue reference and counting four-residue matches. Each search took less than eight hours on an Asus VivoBook 15 laptop. The matches obtained from the two runs were merged, retaining only those smaller matches that were not subsets of any larger match. This process resulted in 133,560 hits. Each residue of a hit was assigned its reference counterpart, from n1 (reference residue 2374) to n6 (reference residue 2379). Among the 284 instances of the HL_85603.2 motif, ARTEM identified 280 complete six-residue matches and three five-residue matches resulting from looped-out n3 residues (Supplementary Fig. S3 A, B, C). The remaining instance was not identified due to an unusual n2 guanosine in *syn* conformation (Supplementary Fig. S3 D, E), with the other five residues matching the reference at an RMSD of 1.535 Å, exceeding the 1.25 Å threshold. To further confirm the optimality of the selected reference instance, we replicated the search using instances of two well-known GNRA tetraloop-like motifs, the UMAC motif^27^ and the UAA/GAN motif^32^, as alternative references and analyzed the RMSD distributions of the identified six-residue matches (Supplementary Fig. S4). The original reference produced a pronounced peak between 0.6 and 1.1 Å, which was absent in both alternatives. Its median RMSD was also notably lower than those of the alternatives: 1.02 Å compared to 1.31 Å and 1.36 Å.

The hits were then annotated with structural features using urslib2^42^, DSSR (version 2.0,^56^), and custom Python scripts. This included per-residue crystal contacts (non-hydrogen atoms from symmetry mates within 4.0 Å), base types of the matched residues, sequence distance between consecutive residues of the match (e.g., 1 for direct neighbors), RNA secondary structure elements (stems and loops) for each residue, and their relationships between consecutive residues (same element, local, long-range), hairpin length and sequence (for hairpin matches), base orientations (anti/syn) and sugar pucker conformations, n2-n5 base pair type according to the Leontis-Westhof (LW) nomenclature^57^, the LW type of the flanking n1-n6 base pair, per-residue information on base-pairing and base-stacking interactions with other residues outside the match, U-turn formation, and Rfam families^45^ and BGSU representative set classes (version 3.370,^46^) of the involved RNA chains.

To account for redundancy, we designed a descriptor string for each hit, which included all the annotated structural features, Rfam families, and BGSU representative set classes. We then merged hits with identical descriptors, keeping the lowest-RMSD hit as the representative. This resulted in a non-redundant set of 23,283 instances (Supplementary Table S1). Subsequently, we merged the instances using descriptors based only on structural features, counting the different Rfam families and BGSU representative set classes annotated for each descriptor, and retaining the lowest-RMSD hit as representative. This produced 13,729 structurally unique hits (Supplementary Table S2). Similarly, we obtained a set of 59 representatives of unique strand topologies (Supplementary Table S3). The use of RNA-focused annotations (Rfam family, BGSU representative set class) was supported by the fact that only 22 of the 23,283 non-redundant hits contained DNA bases (DA, DC, DG, or DT). Missing Rfam/BGSU annotations were treated as a separate class; thus, a motif formally identified in two classes could be an annotation artifact and represent a single instance. Accordingly, we defined a “recurrent” motif as one found in at least three different Rfam families and three different BGSU classes, ensuring that it is represented by at least two unique cases.

No resolution filter was applied to PDB entries in the performed analysis to ensure the inclusion of NMR-derived structures, such as N peptide-BoxB RNA complexes of bacteriophages, which are represented solely by NMR entries in the PDB. To further validate our conclusions, we replicated the entire analysis for PDB entries with resolutions better than 4.0 Å. This subset included 15,463 of the 18,741 PDB entries and 118,788 of the 133,560 matches. We confirmed that it yielded the same set of 59 unique strand topologies and the same 12 recurrent six-residue variants as shown in Fig. 5 (https://github.com/febos/GNRA/tree/main/highres_filter).

A step-by-step description of the performed work is available in the GitHub repository (https://github.com/febos/GNRA/blob/main/REPRODUCE.md).

## Supplementary Information

Below is the link to the electronic supplementary material.

Supplementary Material 1

Supplementary Material 2

Supplementary Material 3

Supplementary Material 4

## Data Availability

The code and data are available at https://github.com/febos/GNRA (10.5281/zenodo.15670527).
