# Supplementary material for: Exploring GNRA tetraloop-like motifs in nucleic acid 3D structures
Source: Sci Rep. 2025 Oct 23;15:37081. doi: 10.1038/s41598-025-21072-9 (PMC12550061; doi:10.1038/s41598-025-21072-9)
Supplement: Supplementary file 1 — Supplementary Material 1 [file 41598_2025_21072_MOESM1_ESM.pdf]

SUPPLEMENTARY MATERIALS

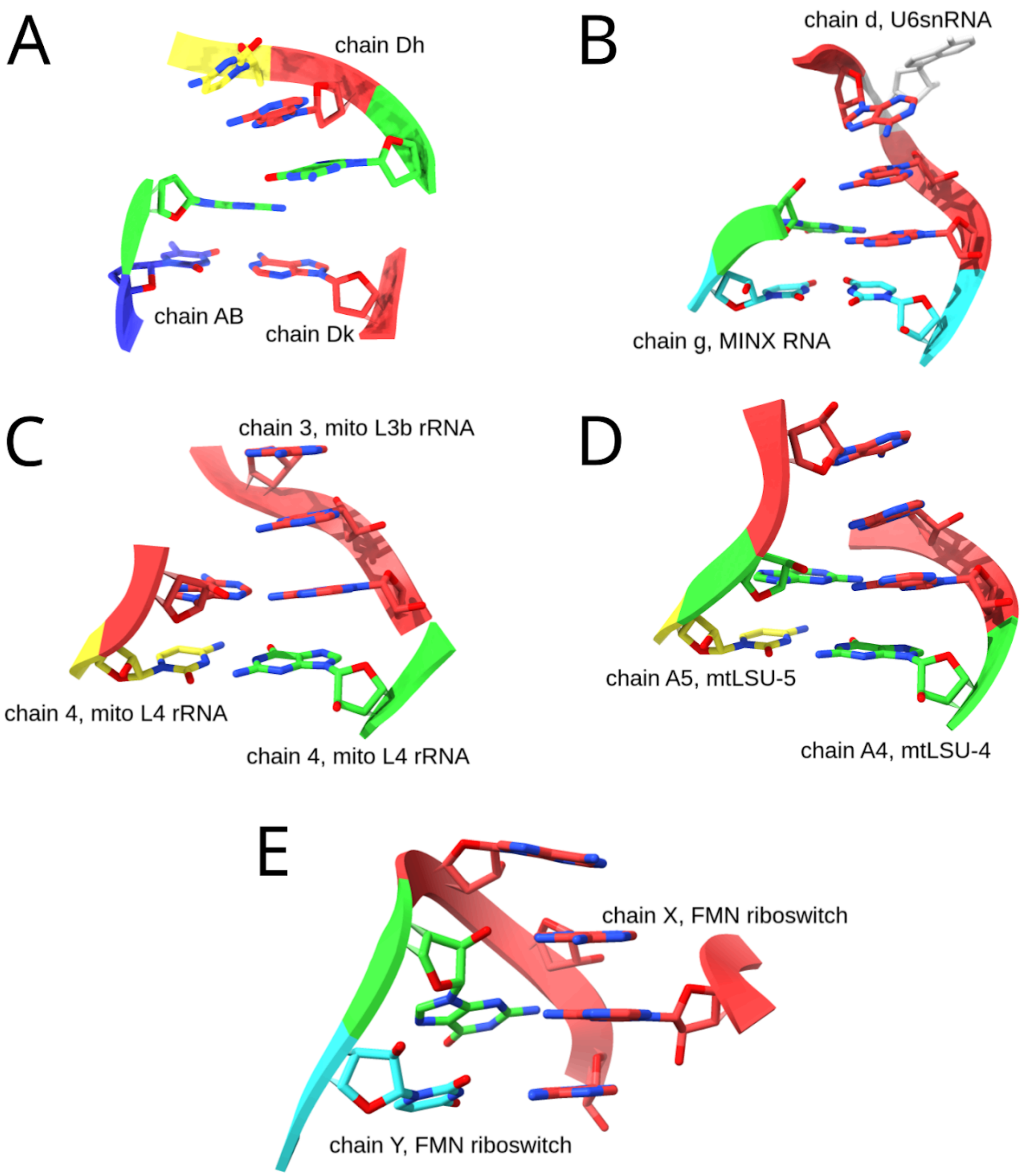

Supplementary Figure S1. Example multi-chain GNRA-like matches. (**A**) PDB entry 7AS5, chain AB, residues 445-446, chain Dh, residues 39-41, chain Dk, residue 9; (**B**) PDB entry 7QTT, chain g, residues 15-16, chain d, residues 27-31; (**C**) PDB entry 7PKT, chain 4, residues 49, 74-75, chain 3, residues 149-151; (**D**) PDB entry 8APN, chain A5, residues 113-115, chain A4, residues 62-64; (**E**) PDB entry 3F4G, chain Y, residues 69-72, chain X, residue 38.

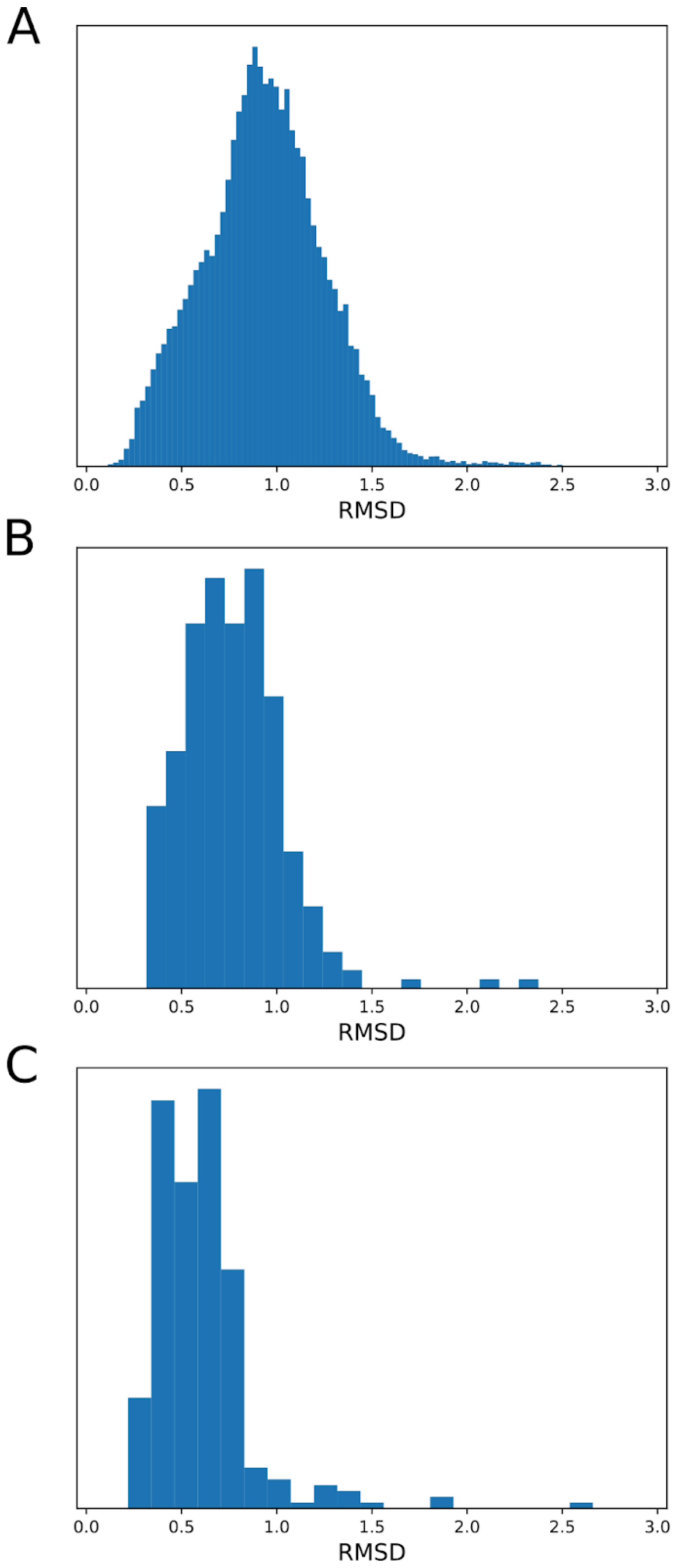

Supplementary Figure S2. Pairwise RMSD values among the 284 GNRA motif instances of the HL_85603.2 motif reported by ARTEM for (**A**) six-residue matches

(GNRA loop and flanking base pair) among all possible pairs, (**B**) six-residue matches with respect to the centroid instance (23S rRNA, PDB entry 8VTW, chain 1A, residues 2374-2379), and (**C**) four-residue matches with the centroid instance (GAAA loop, residues 2375-2378).

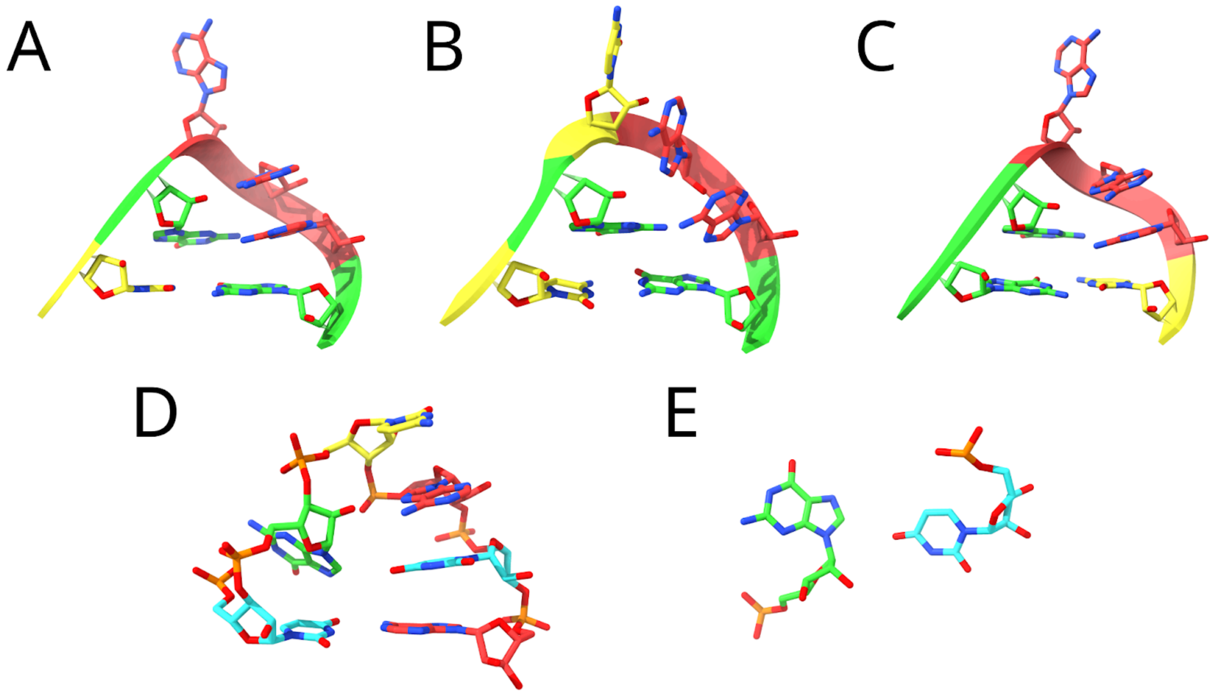

Supplementary Figure S3. Instances of the HL_85603.2 motif with incomplete matches identified by ARTEM. (**A**) Five-residue match, PDB entry 5J7L, chain DA, residues 1806-1811, (**B**) Five-residue match, PDB entry 5J7L, chain DA, residues 1868-1873, (**C**) Five-residue match, PDB entry 3OXE, chain A, residues 49-54, (**D**) Unidentified instance, PDB entry 5VSU, chain I, residues 70-75, and (**E**) its n2-n5 base pair, residues 71 and 74.

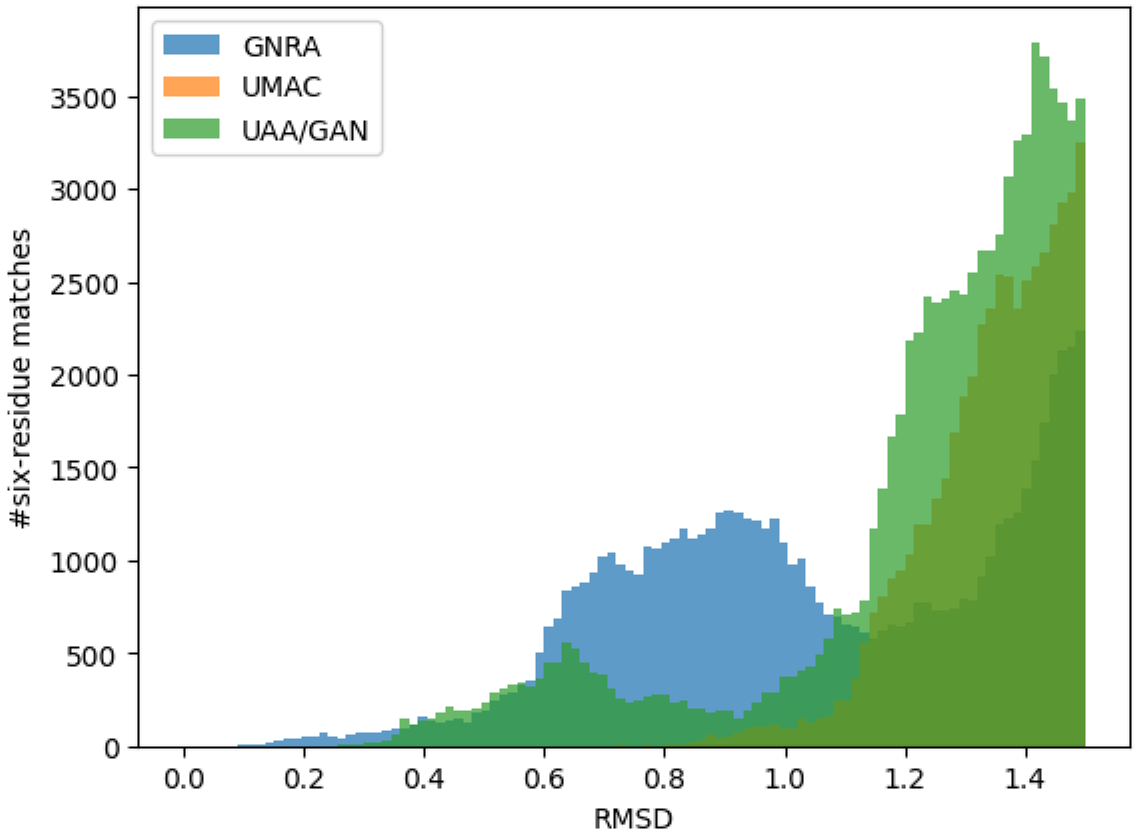

Supplementary Figure S4. RMSD distributions of six-residue matches reported by ARTEM for three alternative references: (i) GNRA tetraloop, median RMSD = 1.02 Å, PDB entry 8VTW, chain 1A, residues 2374-2379; (ii) UMAC tetraloop, median RMSD = 1.36 Å, PDB entry 1NYB, chain B, residues 10-15; (iii) UAA/GAN internal loop, median RMSD = 1.31 Å, PDB entry 4V72, chain BA, residues 1353-1355, 1376-1378.
